# Single-Nucleus Transcriptomics Identifies Neuroblast Migration Programs Sensitive to Reelin and Cannabis in the Adolescent Nucleus Accumbens

**DOI:** 10.1101/2025.04.03.646846

**Published:** 2025-04-03

**Authors:** Yanning Zuo, Numaan Formoli, Avraham Libster, Daimeng Sun, Andrew Turner, Attilio Iemolo, Francesca Telese

## Abstract

The interplay between cannabis exposure during adolescence and genetic predisposition has been linked to increased vulnerability to psychiatric disorders. To investigate the molecular underpinnings of this interaction, we performed single-nucleus RNA sequencing of the nucleus accumbens (NAc) in a mouse model of *Reln* haploinsufficiency, a genetic risk factor for psychiatric disorders, following adolescent exposure to tetrahydrocannabinol (THC), the primary psychoactive component of cannabis. We identified a gene co-expression network influenced by both *Reln* genotype and THC, enriched in genes associated with human psychiatric disorders and predominantly expressed in a GABAergic neuroblast subpopulation. We showed that neuroblasts actively migrated in the adolescent NAc, but declined with age. Cell-to-cell communication analysis further revealed that these neuroblasts receive migratory cues from cholecystokinin interneurons, which express high levels of cannabinoid receptors. Together, these findings provide mechanistic insights into how adolescent THC exposure and genetic risk factors may impair GABAergic circuit maturation.

### Introduction

Adolescence is a critical developmental period during which the brain undergoes critical structural and functional changes essential for adult cognitive and emotional behaviors[1,2]. The enhanced plasticity of the adolescent brain makes it particularly vulnerable to environmental stressors, such as exposure to substances of abuse. Epidemiological research has consistently identified an association between frequent cannabis use and an elevated risk of neuropsychiatric disorders later in life, such as psychosis and substance use disorders[3–7]. While the directionality of the association between adolescent cannabis use and later psychiatric outcomes remains unclear, there is growing consensus that genetic factors contribute to these relationships[8–10].

Preclinical studies have shown that adolescent exposure to tetrahydrocannabinol (THC), the primary psychoactive component of cannabis, leads to deficits in cognitive functions, including working memory and social interactions[11,12]. These behavioral alterations are underpinned by structural and molecular changes induced by THC[13]. For instance, THC exposure during adolescence significantly remodels dendritic structures within the prefrontal cortex[14,15] and persistently alters transcriptional profiles across multiple brain regions, affecting genes critical for synaptic function, neurodevelopment, and metabolism[11,14].

Among the brain regions impacted by adolescent THC exposure[11], the nucleus accumbens (NAc) is particularly significant due its central role in reward processing and motivated behaviors often disrupted by substances of abuse[16–18]. Thus, alterations in gene regulatory networks within cell types of the NAc during this critical developmental period may shape long-term behavioral outcomes[11,19,20]. Recent advances using single-nucleus RNA sequencing (snRNA-seq) have provided valuable insights into cellular diversity and molecular adaptations within the NAc, particularly in the context of substance use[21,22]. For instance, snRNA-seq studies of morphine exposure in rats revealed substantial transcriptional changes in NAc medium spiny neurons (MSNs) affecting synaptic plasticity and neurotransmitter signaling, along with altered myelination in oligodendrocytes[23]. Similarly, snRNA-seq analyses of acute cocaine exposure showed pronounced transcriptional changes in Drd1-expressing MSNs, including induction of immediate early genes[24,25]. However, how specific NAc cell types respond to THC in adolescence and the interplay between these responses and genetic risk factors remain largely unexplored.

One key factor that may influence these outcomes is Reelin, an extracellular matrix protein essential for multiple aspects of brain development and function[26]. Reelin regulates neuronal migration during cortical development, modulates synaptic plasticity, and influences gene expression patterns in both the developing and adult brain [27–29]. Genetic variations in the human RELN gene have increasingly been linked to susceptibility to neuropsychiatric disorders[30,31]. In mouse models, haploinsufficiency of *Reln* has been associated with altered behavioral sensitivity to substances of abuse, including psychostimulants [25,32,33] and synthetic cannabinoids[12,34]. Collectively, these findings suggest that *Reln* deficiency may potentiate the adverse developmental impacts of cannabis exposure during adolescence, providing a compelling framework for investigating the molecular mechanisms underlying its long-lasting neuropsychiatric outcomes.

In this study, we utilized snRNA-seq to investigate the transcriptional consequences of adolescent THC exposure within cell types of the NAc of wild-type (WT) and *Reln* haploinsufficient (*Reln* +/−) mice. We identified specific cell types displaying differential gene expression and distinct gene co-expression networks associated with THC exposure and Reln deficiency. By integrating these transcriptional insights with human genome-wide association study (GWAS) data for substance use disorders and related psychiatric traits, we uncovered molecular pathways connecting THC exposure, *Reln* haploinsufficiency, and elevated neuropsychiatric vulnerability. Our findings provide novel mechanistic insights into cell typespecific responses underlying adolescent cannabis exposure and highlight potential molecular targets for mitigating its adverse long-term effects.

## Results

#### Single-Nucleus RNA Sequencing Reveals Conserved Transcriptomics Cell Types in the Adolescent Nucleus Accumbens

To investigate the molecular consequences of adolescent THC exposure and *Reln* haploinsufficiency in the NAc, we performed snRNA-seq on dissected NAc tissues from mice treated chronically with THC (i.p., 10 mg/kg) or vehicle (**Fig. 1a**). Treatment started at postnatal day 30 (PDN 30), corresponding to early adolescence in mice, and continued daily for 3 weeks. This treatment paradigm was selected based on our prior studies demonstrating that it induces persistent behavioral and transcriptional changes, effectively modeling the long-term impacts of adolescent cannabis exposure[11,12]. Tissues were collected one day after the final injection of THC (PDN-51), and processed via the 10X Genomics droplet-based platform. Our final dataset comprised 5 WT (n=2 vehicle, n=3 THC) and 8 heterozygous *Reln* +/− (n=4 vehicle, n=4 THC) mice (**Supplementary Table 1**). Following quality control filtering (**Fig. S1, Supplementary Table 2**), our integrated dataset included 61,911 high-quality nuclei, clustered into distinct cell populations (**Fig. 1b-c, Supplementary Fig. 2, 3, 4**). Each cluster was annotated based on the expression of canonical marker genes (**Fig. 1d-e, Supplementary Table 3**). Most clusters belonged to the neuronal GABAergic lineage and were characterized by the expression of *Slc32a1* and *Gad1* (**Fig. 1d**). The majority of neurons (>84%) were MSNs identified by the expression of lineage-specification markers such as *Foxp2, Ppp1r1b, and Bcl11b* (**Fig. 1d, Supplementary Fig. 5**). These MSNs were further annotated based on dopamine receptor expression, revealing five distinct clusters of Drd1-expressing MSNs (D1-MSN-1 to D1-MSN-5), three Drd2-expressing MSN clusters (D2-MSN-1 to D2-MSN-3), and a smaller distinct group of Drd3-expressing MSNs (D3-MSN, **Fig. 1d**). Consistent with prior studies[21,22,35,36], these subpopulations formed transcriptional continua marked by well-established subtype markers, such as *Tac1* in D1-MSNs and *Adora2* in D2-MSNs (**Supplementary Fig. 5a)**. Notably, we confirmed the presence of a specialized D1-MSN subpopulation expressing high levels of *Grm8* (D1-MSN-5), a population previously described [37,38] (**Supplementary Fig. 5a**). Additionally, subtype-specific enrichment of genes (e.g. *Ebf1, Onecut2, Sema5b, Greb1l, Htr7*) distinguished these MSN clusters (**Supplementary Fig. 5a**).

Among glial cells, three astrocyte subpopulations exhibited high levels of *Gja1*, two oligodendrocyte populations prominently expressed *Mbp*, and oligodendrocyte precursor cells (OPCs) were marked by *Pdgfra* (**Fig. 1d**). Additional glial populations included *C1qa*-expressing microglia and mural-endothelial cells (Mu-Endo) defined by expression of *Cldn5* and *Vtn* (**Fig. 1d**).

Interneurons (In), defined by *Slc32a1* and *Resp18* expression[21], represented a smaller neuronal fraction, which comprised distinct subtypes of GABAergic neurons expressing unique marker combinations: *Cdh18* in In-1, *St18/Kit* in In-2, *Sst* in In-3, *Vip/Cck* in In-4, and *Nos1/Npy/Sst* in In-5 (**Fig. 1e**). Additionally, we identified a subtype of cholinergic In expressing *Chat* (In-6) (**Fig. 1e**). These interneuron subtypes align well with previously characterized subpopulations in striatal regions, reflecting their functional diversity within the adolescent NAc[39].

As previously reported in the adolescent mouse midbrain[40], we identified a distinct population of proliferating cells characterized by *Top2a* and *Ccnd2* expression, likely neural progenitor cells (NPC, **Fig. 1e**). Additionally, we identified two subsets of GABAergic neuroblasts (NB-1 and NB-2), which expressed immature neuronal markers. The first population retained the expression of neural precursor marker *Igfbpl1* (shared with NPC), while the second exhibited *Calb2* expression, likely marking the initial phases of neuronal differentiation. These findings suggest that the neuroblast categories likely reflect progressive stages of neuronal maturation, rather than distinct or divergent cell lineages.

As previously reported[25,32], *Reln* was primarily expressed in D1-MSNs, specifically the D1-MSN-1 and D1-MSN-2 subtypes. Additionally, *Reln* was expressed in In-3-*Sst*+ and In-4-*Vip+/Cck*+ interneuron subtypes, which also showed high levels of cannabinoid receptor 1 (*Cnr1*) expression (**Supplementary Fig. 5b**). Furthermore, reduced levels of *Reln* were observed in heterozygous Reeler mice, which was consistent with their haploinsufficient genotype (**Supplementary Fig. 5c**).

To assess conservation of NAc cell types and their gene expression signatures across species, we conducted comparative analyses using publicly available human adult NAc snRNA-seq data[41] (**Supplementary Fig. 6**). Statistical comparisons revealed high overall conservation of transcriptional profiles for D1- and D2- MSN populations ($R > 0.5$, $p < 0.05$), while D3-positive MSNs exhibited poor conservation ($R^2 < 0.2$, $p < 0.05$). Glia and interneuron subtypes also demonstrated overall strong cross-species transcriptional conservation ($R^2 > 0.5$, $p < 0.05$). NB subpopulations showed overall poor conservation ($R^2 < 0.3$, $p < 0.05$) but relatively higher correlation with human interneurons than any other cell types, suggesting that they may represent immature interneuron subpopulations. Collectively, these analyses provide a comprehensive cell type atlas of the adolescent NAc in mice and its conservation and divergence from human NAc cell type atlas.

### Distinct Molecular Signatures and Pathway Alterations in the Nucleus Accumbens Induced by THC Exposure and *Reln* Haploinsufficiency

To identify transcriptional alterations induced by adolescent THC exposure and *Reln* haploinsufficiency, we performed differential gene expression (DEG) analysis (**Supplementary Table 4**) comparing THC versus vehicle (**Supplementary Fig. 7)** and *Reln* haploinsufficient (*Reln*-/+) versus WT mice (**Supplementary Fig. 8**). Although THC exposure led to generally more robust transcriptional responses compared to *Reln* haploinsufficiency (**Supplementary Fig. 9a**), a substantial number of DEGs (FDR < 5%, $|z| > 1$) were commonly dysregulated by both conditions1 (**Supplementary Fig. 9b**), suggesting overlapping molecular mechanisms affected by THC and *Reln* haploinsufficiency.

Moreover, among DEGs shared across cell types following THC exposure (**Fig. 2a**), key genes included *Fkbp1a*, gene, encoding for a negative regulator of the mTOR pathway (FKBP12)[42,43], along with genes involved in protein degradation (*Ubb*) and synthesis (*Eif1*), suggesting that THC exposure may lead to enhanced autophagy and altered protein metabolism and, in turn, influence synaptic protein translation and turnover. Consistent with these DEGs, pathway enrichment analyses revealed significant associations with oxidative phosphorylation, retrograde endocannabinoid signaling, addiction pathways, and autophagy (**Fig. 2b, Supplementary Table 5**).

In contrast, DEGs shared across cell types due to *Reln* haploinsufficiency included *Cadm2*, a synaptic cell adhesion molecule associated with lifetime cannabis use and other risky behaviors, reinforcing its role in the genetic predisposition to substance use and related traits[44,45] (**Fig. 2c**). Pathway analysis highlighted significant enrichment for genes related to synaptic transmission, cell-cell adhesion, and nervous system development (**Fig. 2d, Supplementary Table 5**).

Together, these findings illustrate cell type-specific gene expression changes induced by adolescent THC exposure and *Reln* haploinsufficiency in the NAc, many of which were shared across cell types and between conditions.

### Cell Type-Specific Gene Coexpression Networks Reveal THC- and Reln-Associated Modules Linked to Addiction and Psychiatric Disorders

Because genes do not act in isolation but within coordinated networks, we next analyzed gene co-expression to gain a broader view of how adolescent THC exposure and *Reln* genotype shape transcriptional programs. We performed weighted gene coexpression network analysis (WGCNA)[46] adapted for snRNA-seq data[47]. This analysis identified gene coexpression networks, termed modules, which show correlated expression patterns within each cell type (**Supplementary Table 6-7**). Trait-module correlation analysis revealed 43 modules significantly associated with either THC treatment or *Reln* haploinsufficiency (**Supplementary Fig. 10, Supplementary Table 8**), including three that were significantly correlated with both conditions (**Fig. 3a**). The NB-1_*blue* module showed negative correlation with THC treatment and positive correlation with *Reln* genotype, with enriched pathways including axon guidance, transcription regulation, and chromatin remodeling (**Fig. 3b**). In contrast, the D1-MSN-1_*turquoise* module exhibited the opposite pattern, positively correlating with THC and negatively with *Reln* genotype, and was enriched in pathways associated with addiction, nervous system development, and synaptic transmission (**Fig. 3c**). The In-1_*lightcyan* module was negatively correlated with both THC treatment and *Reln* genotype. However, it did not show pathway enrichment, as it primarily consisted of non-coding RNAs without known functional annotation.

To further connect THC- and *Reln*-associated gene networks to human psychiatric risk, we integrated trait-associated modules with human genetic data using the Mergeomics pipeline[48,49]. Specifically, we included cannabis use disorder (CUD)[9], opioid use disorder (OUD)[50], general addiction factor[51], tobacco and alcohol use[52], schizophrenia (SCZ)[53], attention-deficit hyperactivity disorder (ADHD)[54], and externalizing disorders[55] genome-wide association studies

(GWAS). We also used an unrelated GWAS, lean body mass, as negative control[56]. We then mapped the human GWAS signals to genes within 20kb distance of SNPs using brain tissue-specific expression quantitative expression loci (eQTLs) from the GTEx consortium[57]. We leveraged these GWAS-mapped genes to test for enrichment within gene co-expression networks to identify those most likely to be functionally disrupted by genetic risk variants and potentially causal in disease (FDR < 0.05; **Fig. 3d, Supplementary Table 9**). This analysis identified NB-1_*blue* as the top module enriched with genes associated with most traits, including strong enrichments for OUD, tobacco use, and externalizing disorders. Of note, this module was correlated with both THC treatment and *Reln* haploinsufficiency (**Fig. 3a**). Next, we mapped these modules onto Bayesian networks constructed from brain tissues, which comprise gene-gene regulatory relationships that facilitate the prediction of candidate key driver genes[11] (**Supplementary Table 10**). This analysis identified the cannabinoid receptor *Cnr1* among the top key driver genes of NB-1_*blue* module (**Fig. 3e**), suggesting that adolescent THC exposure disrupts the activity of this gene co-expression network via cannabinoid receptors**.**

Together, these findings reveal that THC exposure and *Reln* haploinsufficiency converge on a gene co-expression network in NB-1 neuroblast population, which overlaps with human genetic risk for addiction and psychiatric disorders.

#### Immature Neurons Decline During Development in the Nucleus Accumbens

Our results so far suggest that the NB-1 neuroblast population may represent a critical cellular substrate underlying adolescent brain vulnerability. Moreover, the conservation analysis comparing NB-1 neurons to human adult NAc revealed that among neuronal cell types, NB-1 cells exhibited modest conservation exclusively with interneurons, and not MSNs (**Fig. S6**), reinforcing their classification as immature interneurons. To further elucidate their developmental trajectory, we next examined the conservation of mouse neuroblasts across multiple developmental stages. Therefore, we performed conservation analysis between our adolescent mouse NAc interneurons dataset and publicly available snRNA-seq datasets from the juvenile mouse striatum[40] and adult mouse NAc[21]. Adolescent NB-1 neurons showed conservation with neuroblasts populations detected within the juvenile dataset (NB, $R^2 > 0.5$, $p < 0.05$, **Fig. 4a**) and the adult mouse NAc dataset NB-2 ($R^2 = 0.42$, $p < 0.05$, **Fig. 4b**). These findings reinforce the interpretation of NB-1 neurons as an immature interneuron population in the postnatal NAc.

We next used Monocle3 pseudotime analysis[58] to reconstruct the differentiation trajectory of NB-1 within the interneuron lineage. Consistently with their immature molecular signature, this analysis confirmed a developmental hierarchy where NB-1 neurons occupied the earliest pseudotime stage (**Fig. 4c-d**). This analysis further predicted that NB-1 neurons transitioned through another immature population (NB-2, expressing *Calb2*) before reaching a transitional subtype (In-1, expressing *Cdh18*) and subsequently bifurcating into mature interneuron lineages. One branch led to mature In-2-*St18*+ parvalbumin population and In-6-*Chat*+ cholinergic population, while the other branch gave rise to In-3-*Sst+,* In-4-*Cck*+, *and* In-5-*Nos1*+ In subtypes. As expected, peak expression of NB-1 makers (*Igfbpl1*) occurred early in pseudotime, followed by the peak expression of NB-2 (*Calb2)* and transition In-1 subtype (*Cdh18*), whereas mature In markers peaked later in pseudotime with distinct patterns (**Fig. 4e)**.

We validated this presence of neuroblasts in the NAc through direct immunochemistry quantification of doublecortin (DCX)-positive cells using tissue sections from adolescent (PND 35-50) and adult (PND 120) mice (**Fig. 5a-c**). DCX is a canonical marker of migrating neuronal precursors during both embryonic and postnatal brain development, particularly within canonical neurogenic niches such as the subventricular zone (SVZ) and dentate gyrus (DG)[59,60]. DCX immunostaining revealed that $DCX^+$ cells appeared elongated and aligned along a defined path extending from the lateral ventricle (LV) into the NAc, consistent with active migration (**Fig. 5a, b**). High-magnification imaging confirmed cells with migratory morphology, including leading processes characteristic of migrating neuroblasts (**Fig. c**). To quantify these differences, we developed a customized image analysis pipeline in CellProfiler[61] to accurately segment cell body-like fluorescent signals in brain tissue sections, significantly improving detection accuracy compared to baseline methods (**Supplementary Fig. 11**). Using this pipeline, we found a significant decline in the proportion of $DCX^+$ neurons from 2.4% in adolescence to 1.2% in adulthood (**Fig. 5d**).

To investigate how adolescent THC exposure and *Reln* haploinsufficiency may alter signaling to NB-1 neurons, we performed cell-to-cell communication analysis using LIANA, a computational framework that integrates multiple ligand–receptor inference methods[62]. We specifically focused on In-4 interneurons which co-express *Reln* and notably high levels of *Cnr1* (**Supplementary Fig. 5**), and therefore, serving as a potential hub for mediating THC-dependent signaling to neuroblasts during adolescence. This analysis revealed significant enrichment of ligand-receptor interactions implicated in neuronal migration and axon guidance (**Fig. 5e, Supplementary Table 11**). Notably, several ligand-receptor pairs are known to be involved in axonal growth and cell migration, including *Igf1-Igf1r*[63], *Slit1–Robo2*[64,65], *Reln-Lrp8*[66], *Fgf13–Fgfr2*[67], and *Sema6a-Plxna4*[68], suggesting that altered Reelin signaling and adolescent THC exposure may have an impact on migratory cues in the adolescent NAc.

To further delineate the regulatory mechanisms by which adolescent THC exposure and *Reln* haploinsufficiency influence NB-1 neuronal maturation, we performed gene regulatory network analysis using SCENIC[69], focusing on In-4 interneurons (**Supplementary Table 12**). This analysis identified the *Nrf21* (COUP-TF1) regulon as a key transcriptional program defining the In-4 interneurons cell state (**Fig. 5f**). Notably, COUP-TF1 is a transcription factor of the nuclear receptor family that is required for the migration of cortical interneurons and the proper formation of the cortical local inhibitory circuitry[70]. Moreover, the gene regulatory network analysis revealed that *Sema3ac* and *Igf1*, both implicated in neuroblasts migration, are predicted targets of *Nr2f1* and cell type markers for In-4 interneurons, suggesting that *Nr2f1* may orchestrate a transcriptional program in these cells that supports neuroblast migration and neuronal maturation in the adolescent NAc.

Collectively, our findings provide novel insights into the developmental regulation of neuroblasts by extrinsic factors expressed by cannabinoid-responsive GABAergic cells in the adolescent NAc.

## Discussion

This study provides the first comprehensive single-nucleus transcriptomic atlas of the mouse adolescent NAc, a central brain region implicated in reward, motivation, and addiction. By leveraging snRNA-seq, we identified cell type-specific transcriptional signatures associated with adolescent THC exposure and *Reln* haploinsufficiency. Our findings advance the understanding of how these environmental and genetic risk factors disrupt molecular pathways during a key brain developmental window.

A critical finding of this study is the identification and molecular characterization of NB-1 GABAergic neuroblasts in the adolescent NAc, a finding independently validated through quantitative analysis of DCX protein expression. Our results are consistent with previous reports of postnatal neurogenesis in the mouse NAc[71]. Extending these observations, our study provides further direct evidence of active migration of DCX-positive GABAergic neuronal precursors within the adolescent NAc and novel insights into the molecular mechanisms underlying their function.

Our cell-to-cell interaction analysis from In-4 interneurons to NB-1 neuroblasts identified several ligand-receptor pairs with well-established roles in neuronal migration and niche regulation, particularly in canonical adult neurogenic regions[72,73]. Among these, *Igf1-Igfr1* interaction emerged as the top-ranked pair, implicating insulin-like growth factor (IGF-1) signaling as a major regulatory axis. IGF-1 has well-established roles in neuronal differentiation and survival, but emerging evidence also supports its involvement in neuroblast migration and positioning in the SVZ[63]. In addition, the identification of *Reln-Lrp8* interaction reinforces the hypothesis that Reelin signaling contributes to adolescent neuronal migration in a manner analogous to its well-established role in embryonic cortical layering[26,74]. Of note, while Reelin signaling has been implicated in adult neurogenesis in the DG[75], its role in postnatal neurogenesis in the SVZ has not been explored. Together these findings support a model in which cannabinoid-responsive In-4 interneurons may contribute to the migration and maturation of NB-1 neuroblasts through a coordinated network of migratory cues, which may be sensitive to disruption by adolescent cannabis exposure, and genetic perturbations, such as *Reln* haploinsufficiency. Supporting this interpretation, our key driver analysis identified *Cnr1* as a critical upstream regulator of the NB-1_*blue* gene co-expression network.

The integration of these cell type-specific findings with human GWAS data highlights the broader translational relevance of our discoveries. Specifically, the NB-1_*blue* gene co-expression network, associated with both THC exposure and *Reln* deficiency, exhibited significant enrichment for genetic variants linked to SUD and externalizing behaviors. Given the proposed role of NB-1 in GABAergic neuronal maturation in the adolescent NAc, disruption of this pathway may predispose individuals to altered reward circuit function and heightened addiction vulnerability.

However, several limitations should be considered when interpreting our results. First, although our study provides evidence for the developmental trajectory of NB-1 neurons, our snRNA-seq data represents a snapshot captured at a single timepoint. Therefore, the functional roles and ultimate fate of these cells remain unclear. Future studies employing lineage tracing, electrophysiological recordings, or functional imaging will be necessary to clarify their specific contributions to circuit maturation and behavior. Additionally, while inference methods suggest a role for specific migratory signaling influencing the migration of neuroblasts, the molecular mechanisms governing these processes have yet to be fully elucidated, warranting additional studies into manipulating potential regulators and environmental factors influencing these processes during adolescence.

In conclusion, our findings enhance our understanding of how adolescent cannabis use may alter the maturation of reward circuitry, with significant implications for vulnerability to psychiatric disorders later in life.

## Materials and Method

#### Animals

All experimental procedures were approved by the institutional animal care and use committee at the University of California, San Diego. Mice were housed (3-4 per cage) under a 12h light/12h dark cycle and provided with food and water ad libitum.

For snRNA-seq experiments, the heterozygous, haploinsufficient reeler mouse[26] (*Reln* -/+) were bred in house using the B6C3Fe a/a-Relnrl/J line (The Jackson Laboratory, #000235). For IHC experiments, n=12 C57BL/6J male mice were purchased from Jackson Laboratory (#000664).

#### Drug treatment protocol and experimental design

THC was supplied by the U.S. National Institute on Drug Abuse and prepared in a vehicle solution containing ethanol, tween, and 0.9% saline in a ratio of 1:1:18. The solution was freshly prepared on the day of administration. Specifically, we administered vehicle or THC (10 mg/kg) daily to mice by intraperitoneal injections in the early adolescent period, from PND 30 to PND 50 +/- 3 days, as previously reported[11,12,76]. In our previous studies[11,12], we employed the same drug treatment protocol and demonstrated that it resulted in long term behavioral deficits and plasma THC concentrations of 5–6 ng/ml one day after the final injection. While data on plasma THC levels in adolescent users remain limited, these findings align with studies on frequent adult cannabis users, which reported median plasma THC levels of ≥5.0 ng/ml for up to three days of abstinence[77–79]. Despite the variability in THC content across cannabis products used by humans, the dosage used in this study is likely reflective of heavy cannabis use. In this study, we collected the brain tissues 1 day after the last injection of THC. We used 5 WT mice (n= 2 vehicle, n=3 THC), and 8 *Reln* -/+ mice (n=4 vehicle, n=4 THC).

#### Single-nucleus RNA-seq data generation

Brains were sliced on a cryostat and microdissected by punching the NAc starting at these coordinates: +1.78. Single nucleus RNA-seq libraries were performed by the UC San Diego Center for Epigenomics, using the Droplet-based Chromium Single-Cell 3' solution (10x Genomics, v3 chemistry). Briefly, tissue punches from individual mice were homogenized via glass dounce. Nuclei were then resuspended in 500 μL of nuclei permeabilization buffer (0.1% Triton-X-100 (Sigma-Aldrich, T8787), 1X protease inhibitor, 1 mM DTT, and 1U/μL RNase inhibitor (Promega, N211B), 2% BSA (Sigma-Aldrich, SRE0036) in PBS). Samples were incubated on a rotator for 5 min at 4°C and then centrifuged at 500 rcf for 5 min (4°C, run speed 3/3). Supernatant was removed and pellet was resuspended in 400 μL of sort buffer (1 mM EDTA 0.2 U/μL RNase inhibitor (Promega, N211B), 2% BSA (Sigma-Aldrich, SRE0036) in PBS) and stained with DRAQ7 (1:100; Cell Signaling, 7406). 75,000 nuclei were sorted using a SH800 sorter (Sony) into 50 μL of collection buffer consisting of 1 U/μL RNase inhibitor in 5% BSA; the FACS gating strategy sorted based on particle size and DRAQ7 fluorescence. Sorted nuclei were then centrifuged at 1000 rcf for 15 min (4°C, run speed 3/3) and supernatant was removed. Nuclei were resuspended in 35 μL of reaction buffer (0.2 U/μL RNase inhibitor (Promega, N211B), 2% BSA (Sigma-Aldrich, SRE0036) in PBS) and counted on a hemocytometer. 12,000 nuclei were loaded onto a Chromium Controller (10x Genomics). Libraries were generated using the Chromium Single-Cell 3′ Library Construction Kit v3 (10x Genomics, 1000075) with the Chromium Single-Cell B Chip Kit (10x Genomics, 1000153) and the Chromium i7 Multiplex Kit for sample indexing (10x Genomics, 120262) according to manufacturer specifications. CDNA was amplified for 12 PCR cycles. SPRISelect reagent (Beckman Coulter, B23319) was used for size selection and clean-up steps. Final library concentration was assessed by Qubit dsDNA HS Assay Kit (Thermo-Fisher Scientific) and fragment size was checked using Tapestation High Sensitivity D1000 (Agilent) to ensure that fragment sizes were normally distributed about 500 bp. Libraries were sequenced using the NextSeq500 and a NovaSeq6000 (Illumina) with these read lengths: 28 + 8 + 91 (Read1 + Index1 + Read2).

#### Raw data processing

Raw sequencing data was processed with cellranger version 6.0.2 and aligned to mm10-3.0.0. Gene expression matrices from 13 samples were integrated using the aggregation function aggr in cellranger 6.0.1 to merge into one gene expression matrix for the following data analysis. In total, 82251 nuclei were detected in our raw data.

#### Quality control and doublet removal

The merged gene expression matrix was imported into the R package Seurat[80] version 4.9.9.9058 using functions `Read10X_h5()` and `CreateSeuratObject()`. Nuclei with the following parameters were kept for further analysis: RNA count between 1000 and 16000; gene/non-coding RNA number between 500 and 6000; percentage of mitochondrial genes less than 2%; percentage of small ribosomal subunit (RPS) genes less than 1% and percentage of large ribosomal subunit (RPL) genes less than 1%.

The R package DoubletFinder[81] version 2.0.3 was used to detect potential doublets in each sample using a custom script. Dimensionality reduction was performed on each sample using the first 20 principal components (PCs). A default setting of 0.25 generated an artificial doublet percentage (pN). Principal component neighborhood size (pK) was determined by using the functions `paramSweep_v3()`, `summarizeSweep()`, and `find.pK()`. We assume that 5% of the total nuclei were doublets. 68917 nuclei remained after quality control and doublet removal.

**Single-nucleus transcriptome processing, cell clustering, and cell annotation**
`SCTransform()` in Seurat based on regularized negative binomial regression was used for normalization and removal of variance due to mitochondrial gene percentage, sequencing depth (RNA counts), and RPL and RPS gene percentage. Dimensional reduction was performed using `RunPCA()` in Seurat, and the first 50 PCs were used for nuclei clustering and visualization using `FindNeighbors()`, `FindClusters()`, and `RunUMAP()`. A resolution of 0.7 was used for `FindClusters()`. Markers for each nuclear cluster were identified using `FindMarkers()` in Seurat. We used a list of manually curated canonical markers to facilitate cell type annotation: Neuron: Snap25, Syt1; Glutamatergic excitatory neuron: Slc17a7; GABAergic interneuron: Resp18, Gad1; Medium spiny neurons neuron (MSN): Ppp1r1b, Foxp2, Bcl11b; Drd1+ medium spiny neuron: Drd1, Pdyn, Tac1; Drd2 MSN: Drd2, Adora2a, Penk; Oligodendrocyte: Mbp; Oligodendrocyte precusor cell: Pdgfra; Astrocyte: Gja1; Neural stem cell and neuroblast: Top2a; Microglia: C1qa; Mural cell: Vtn; Endothelial cell: Cldn5. Two additional clusters were excluded due to high levels of mitochondrial genes and a mixed expression of oligodendrocyte and neuronal markers. We further excluded glutamatergic excitatory neuron clusters because, according to the literature, NAc does not contain glutamatergic excitatory neurons. A total number of 61911 nuclei were used for all downstream analyses.

**Differentially expressed gene identification**
We used the `FindMarkers()` function in Seurat to identify differentially expressed genes for treatment (THC versus vehicle) and genotype (*Reln* -/+ versus WT) in each cell cluster. An expression fold change of 1.2 (the absolute value of avg_log2FC larger than 0.25) with a false discovery rate less than 0.05 was used as the significant threshold.

**Pathway annotation**
Pathway annotations in our study were performed using the enrichR R package[82]. The following enrichR databases were used: KEGG_2021_Human, GO_Biological_Process_2021, BioCarta_2016, and Reactome_2022. Pathway enrichment was calculated based on the deviation from the expected rank for terms, and P-values were adjusted for multiple testing corrections using the Benjamini-Hochberg correction. Adjusted P values of 0.05 were considered significant.

**WGCNA**
Raw snRNA-seq counts were normalized with Seurat normalization `NormalizeData()` and then log-transformed, and genes with a standard deviation larger than 0 were chosen for WGCNA[46] analysis adapted for snRNA-seq[47]. The WGCNA R package was used to generate signed co-expression networks for each cell subtype separately.
To generate cell subtype-specific gene coexpression networks, we selected high-quality nuclei based on the RNA count. For each cell subtype, we ranked the RNA count captured in each nucleus. We selected the top 5%-40% nuclei based on the RNA count of their corresponding cell subtype. This filtering removed nuclei having excessive RNA counts, which might indicate potential doublets, and those with low RNA counts, which indicate potential high drop-out rates. An adjacency matrix was generated to capture pair-wise correlation relationships computed by biweight midcorrelation between genes, using cell subtype-specific soft power parameters for the best fit of a scale-free topology. A topological overlap measure (TOM) matrix was then calculated based on the adjacency matrix to measure the relative interconnectedness between each pair of genes. Next, a gene dendrogram was built using the flashClust function based on hierarchical clustering. Branches of the dendrogram correspond to clusters of positively correlated genes, which are termed modules. `cutreeDynamic()` based on a branch-cutting algorithm was used to identify modules using the following parameters: method = hybrid, minModuleSize = 30, pamStage = F, pamRespectsDendro = T, deepSplit = 2, and cutHeight = 0.9999. After module detection, module eigengenes (MEs) were calculated as the first principal component of genes in a module to summarize each module's expression profile. Modules with ME correlation higher than 0.85 were considered highly similar modules and merged using the mergeCloseModules function.

**Trait module correlation**

To identify module-trait associations, Pearson's correlation between module MEs, THC treatment, and Reln genotype was calculated. Modules with significant correlation ($p < 0.05$) with one or more traits were considered relevant. Z-scores of Pearson's correlation across all the modules of a cell subtype were calculated to facilitate visualization.

**Associating WGCNA modules with human GWAS traits**

To test whether the WGCNA modules were enriched in genes implicated in cannabis use-related human GWAS traits, we used the Mergeomics[48] R package that integrates gene sets with GWAS summary statistics through functional genomics. The following GWAS summary statistics were used in our study:
- Cannabis use disorder (CUD) [9] from https://www.med.unc.edu/pgc/download-results/.
- Opioid use disorder (OUD) [50] from https://www.med.unc.edu/pgc/download-results/.
- General addiction factor[51] via personal communication.
- Tobacco and alcohol use traits [52] from https://conservancy.umn.edu/handle/11299/241912.
- Schizophrenia (SCZ) [53] from https://www.med.unc.edu/pgc/download-results/.
- Attention-deficit hyperactivity disorder (ADHD)[54] from https://www.med.unc.edu/pgc/download-results/.
- Externalizing factor[55] via personal communication.
- Lean body mass (LBM)[56] from http://www.gefos.org/?q=content/adult-lean-mass-gwas-2017.

SNPs were adjusted for linkage disequilibrium with a cutoff of 0.5 to remove redundant SNPs. GWAS SNPs were mapped to genes using the following mapping strategies: (1) genes within 20kb of SNPs and (2) genes under the regulation of the NAc tissue-specific expression and splicing quantitative trait loci (eQTLs/sQTLs) from GTEx[83] v8 for human NAc. GWAS SNPs, their mapped genes, and the corresponding GWAS p values were then intersected with co-expression modules for each brain region using the Marker Set Enrichment Analysis (MSEA) function in Mergeomics to assess whether co-expression modules, compared to randomly shuffled gene sets of the same size, were enriched for genes with low p-value SNPs. Modules with an FDR < 0.05 were considered significantly enriched for GWAS-related genes.

**Weighted Key Driver Analysis (wKDA)**

wKDA from the Mergeomics[48] R package was used to identify key drivers of selected modules or DEGs based on a gene interaction network. We used a brain-specific Bayesian network capturing causal gene-gene regulatory relationships constructed using the RimbaNet package and numerous human and mouse brain genetic and transcriptomic datasets previously used in our study[11]. In wKDA, key driver genes were first identified based on the input network's topology. Subsequently, the input genes were overlaid onto the network. The key driver genes whose downstream network neighbors were enriched with the input genes were highlighted as key drivers for the trait-related genes or modules. The parameters used were as follow: edgefactor = 1 (considering edge weights), depth = 1 (searching depth), direction = 0 (not considering the directions of the interactions), nperm = 10000 (permutation numbers), ndrivers = 10 (up to 10 key drivers from each input gene list). Networks of prioritized key drivers were visualized using Cytoscape version 3.8.228.

**Trajectory analysis**

R package Monocle3[84] was used to infer the pseudotime trajectory of neural progenitor cells and interneuron subtypes. UMAP dimensional reduction of the selected cell subtype subset was performed using the first 30 principal components. Function `learn_graph() was used to learn the projection space graph and `order_cells` was applied to assign inferred pseudotime values of each nucleus, with the root cell population set as neural progenitor cells. To visualize gene expression level change along the pseudotime, a Generalized Additive Model (GAM) smoother was applied using geom_smooth() with the formula y ~ s(x, k = 30, bs = "cs").

**Conservation Analysis**

To compare the similarity of cell types across species and studies, cell type specific t-statistics for each gene were calculated on SCTransformed expression values using the formula below following previously reported methods[85]:

$$t_i = \frac{\sqrt{n_x} \cdot (\mu_x - \mu_y)}{s_{pooled}}$$

Where

$$\mu_x = \underline{X} = \frac{\sum_{i=1}^{n_x} X_i}{n_x}$$

$$\mu_y = \underline{Y} = \frac{\sum_{i=1}^{y} Y_i}{n_y}$$

$$s_{pooled} = \sqrt{\frac{{s_x}^2 + {s_y}^2}{2}}$$

$X_i$ denotes the expression values of gene $i$ in a specific cell type $x$, with $\mu_x$ being the mean expression value; $Y_i$ denotes the expression values of gene $i$ in all other cells, with $\mu_y$ being the mean expression value; $n_x$ and $n_y$ denotes the number of cells in the specific cell type $x$ and that of all other cells, respectively; Similarly, $s_x$ and $s_y$ denotes the standard deviation of gene $i$ expression in a specific cell type $x$ and that of all other cells, respectively.

Adult mouse NAc cell types from Chen et al[21], adult human NAc cell types from Tran et al[41], and juvenile mouse dorsal striatum cell types from Zeisel et al.[40] were used to compare with our adolescent mouse cell types. For Chen et al, cell type identities identified in the study were used and SCTransform was applied, regressing out reading depth differences across cells. T-stats were calculated following the same formula on SCTransformed expression values. The same procedure was performed on Tran et al followed by gene symbols converted from human to mouse, with the exception of further regressing out variances from donor, sex, processBatch, percent.RPL and percent.RPS. 5 cell populations "drop.doublet_A", "drop.doublet_B", "drop.doublet_C", "drop.doublet_D", "drop.lowNTx" with bad quality identified in Tran et al were also removed before t-stats calculation For Zeisel 2018, because the original cell type identities were not detailed for our analysis, clustering and cell annotation were performed on high-quality cells using the first 30 principal components and t-stats were calculated using SCTransformed expression. The quality control parameters are as follows: nFeature_RNA > 500, nFeature_RNA < 6000, nCount_RNA > 1000, nCount_RNA < 16000, percent.mt < 2 (n = 11775 before QC, n = 8788 after QC). percent.mt and nCount_RNA were regressed out during SCTransform.
After the calculation of t-stats, Pearson's correlation and its corresponding p value were calculated using `cor.result` using the top 100 upregulated genes from each cell type.

**Cell-to-cell Communication Analysis**
R package LIANA[86] (version 0.1.14) was used to identify cell-to-cell communication between the cell subtypes we identified. LIANA's consensus resource was converted to murine symbols using select_resource(), and the orthologous resource was generated using generate_homologs() with the target organism being mouse. LIANA was then run with liana_wrap() and results were aggregated using liana_res(). Results with an aggregate rank equal to or smaller than 0.01 were considered significant and were visualized using liana_dotplot().

**Gene Regulatory Network Analysis**
R package SCENIC[69] (version 1.3.1) was used for transcriptional factor inference. Gene motif information was downloaded from "https://resources.aertslab.org/cistarget/databases/old/mus_musculus/mm9/refseq_r45/mc9nr/gene_based/mm9-500bp-upstream-7species.mc9nr.feather" and "https://resources.aertslab.org/cistarget/databases/old/mus_musculus/mm9/refseq_r45/mc9nr/gene_based/mm9-tss-centered-10kb-7species.mc9nr.feather". Organism was then set to "mgi" for mouse and the database directory was set to "cisTarget_databases".
First, Genie3 gene regulatory networks (GRNs) were calculated using raw gene expression matrices. Raw gene expression matrices were filtered with `geneFiltering()` to keep genes with a minimal count of 3 in at least 1% of all the input cells. Positive or negative associations between the transcriptional factor and potential targets were calculated through

`runCorrelation()`. Then Genie3 GRNs were calculated using log2-normalized matrices by running `runGenie3()`.
Then, the GRNs were built and scored using `runSCENIC_1_coexNetwork2modules()`, `runSCENIC_2_createRegulons()`, and `runSCENIC_3_scoreCells()`. Regulon information was extracted using `get_regulons()`, `regulonsToGeneLists()`, `get_regulons_AUC()`, `get_regulon_thresholds()`, and `get_embeddings()`. Cell-type-specific regulon activities were calculated using the AUC of each regulon and visualized using `ComplexHeatmap::Heatmap()`. In addition, regulon specificity scores (RSSs) were calculated to identify cell-type-specific transcriptional factors using `calcRSS()`.

**Immunohistochemistry and Image Acquisition**

Mouse brain tissues were collected during adolescence (PDN 35-55, n=8) and adulthood (PDN 120, n=4) after transcardial perfusion with 4% paraformaldehyde (PFA) (Millipore Sigma, 441244). Brains were kept in 4% PFA for 24 hours at 4℃, followed by dehydration in 30% sucrose. 16μm coronal sections were obtained using a microtome and stored at -20℃ in cryoprotectant solution. Brain sections (bregma point 1.4 - 0.7) containing the NAc were selected for IHC using anti-DCX (Cell Signaling, 4604S) as primary antibody and donkey anti-rabbit (Invitrogen, 21206) as secondary antibody. After three PBS washes, sections were mounted on slides, dried at 60℃ for 1 hr, and incubated with a blocking buffer (0.3% Triton X, 10% NGS in PBS) for 90 min at RT. Primary antibodies diluted in the antibody dilution buffer (0.3% Triton X, 2% NGS in PBS) were applied overnight at 4℃. Slides were then washed in PBS, incubated with secondary antibodies for 1.5 hr in darkness at RT, stained with 1200 nM DAPI for 10 min, air-dried, and stored at 4℃ for microscopy. All images were acquired using the Keyence BZ-X810 All-in-One wide-field fluorescence microscope. Z-stacks of NAc were obtained at 20x magnification using an air immersion objective lens (PlanFluor 20x, NA 0.45; Nikon).

**Fluorescence Image analysis**

Automated nuclei counting was performed using a customized CellProfiler[61] (V4.2.5) pipeline. IHC images of mouse NAc were loaded into the pipeline and converted into grayscale images using the 'ColorToGray' module. Noise reduction was first performed on each image using the 'CorrectIlluminationCalculate' module and the 'CorrectIlluminationApply' module. Method 'Background', a block size of 60 pixels, and other parameters as default were employed in the 'CorrectIlluminationCalculate' module to calculate background intensity. Subsequently, the calculated background intensity was subtracted in the 'CorrectIlluminationApply' module. Next, the number of all captured cells was determined from the noise-reduced DAPI images of each brain section using the 'Watershed' module with advanced settings. Footprint of 4, smoothing factor of 2, minimum distance between seeds of 2, and other parameters as default were employed in the 'Watershed' module to identify each DAPI signal as individual objects resembling nuclei. Sizes of identified nucleus were measured using the 'MeasureObjectSizeShape' module and identified nucleus were subsequently filtered by the 'FilterObject' module, using filtering method of 'Limit', category of 'AreaShape', and measurement of 'MaxFeretDiameter' and 'MinFeretDiameter'. Positive nucleus objects were defined as objects with a 'MaxFeretDiameter' smaller than 26 pixels and a 'MinFeretDiameter'

greater than 8 pixels. Objects outside of this criteria were discarded. DCX signal was determined from the noise-reduced fluorescent images using the 'IdentifyPrimaryObjects' module with advanced settings. DCX-positive cells were defined as objects with a diameter between 5 to 15 pixels. Objects outside of this range were discarded. A 'Global' 'Robust Background' threshold was used to assign pixels based on the distribution of pixel intensity. After excluding 5% of the dimmest and brightest pixels, the median and standard deviation were calculated from the remaining pixels. The threshold was set as 5 standard deviations above the median. No smoothing and declumping were employed, and all other settings were as default. DCX+ cells were identified using the 'Relate Objects' module. DCX+ objects were set as parents and positive DAPI objects were set as children. Overlap between parent and child objects was examined. DAPI objects related to DCX objects were saved as a new object set. All cells in the new object set were positive for DCX. Cell profiler measurements, including numbers of identified DCX+ cells and DAPI objects, were output to a .csv file using the 'ExportToSpreadsheet' module. The susceptibility to under-segment dense images was tested between our customized CellProfiler pipeline and a baseline CellProfiler pipeline.

**Statistical analysis**
Statistical analysis was performed using R. For the customized cell profiler pipeline benchmarking analysis, Welch's two-tailed, unpaired t-test was performed to assess the difference in performance between the customized CellProfiler pipeline and baseline pipeline under the same cluster probabilities. For the DCX IHC analysis, an unpaired t-test was performed to assess the difference in the number of DCX+ cells in the NAc of adolescent mice and adult mice. $P \leq 0.05$ was defined as statistically significant.

## Figure Legends

**Figure 1. snRNA-seq profiling of adolescent NAc identifies diverse neuronal and non-neuronal cell types. a)** Experimental timeline and workflow. THC or vehicle treatments were administered daily from PND30–50 (adolescence). NAc tissue was collected on PND51 and processed via snRNA-seq (10X Genomics). **b)** UMAP visualization of 61,911 integrated nuclei, colored by annotated cell clusters. **c)** Bar plot indicates the number of nuclei per identified cluster. **d)** Violin plots depicting expression levels of selected marker genes across annotated clusters, highlighting main cell type markers. Expression values shown as normalized RNA counts. **e)** Violin plots depicting expression levels of selected marker genes across interneurons (In), neuroblasts (NB) and neural progenitor cells (NPC). Expression values shown as normalized RNA counts.

**Figure 2: Differential gene expression and pathway analysis in the NAc induced by adolescent THC exposure and Reln haploinsufficiency. a)** Volcano plot showing shared DEGs across cell types induced by THC exposure **b)** Pathway enrichment analysis for THC-induced DEGs across neuronal cell types. **c)** Volcano plot of shared DEGs across cell types resulting from *Reln* haploinsufficiency. **d)** Pathway enrichment analysis for DEGs due to *Reln* haploinsufficiency.

**Figure 3: Cell type-specific gene co-expression network analysis in the adolescent NAc. a)** Heatmap of module-trait correlations between identified gene modules and experimental conditions (THC treatment, *Reln* haploinsufficiency). Color depicts the correlation coefficient *p < 0.05; ***p < 0.001. **b)** Pathway enrichment analysis for the NB-1_*blue* module. **c)** Pathway enrichment analysis for the D1-MSN-1 turquoise module. **d)** Heatmap of modules enriched in GWAS-related genes from Mergeomics. Color depicts enrichment p-values as -log10(FDR). Adjusted *p < 0.05; **p < 0.01; ***p < 0.001. **e)** Visualization of the Bayesian network shared by the NB-1_*blue* module. Diamond nodes are key driver genes; hexagon nodes are hub genes; blue nodes are genes in the NB-1_*blue* module; green nodes are genes downregulated by THC; grey nodes are genes in the BN but not in the aforementioned categories.

**Figure 4: NB-1 is an immature GABAergic interneuron subtype. a)** Heatmap of Pearson correlation values evaluating the relationship between our adolescent mouse-derived NAc GABAergic neurons (columns) versus the inhibitory neurons populations, derived from mouse PDN 12-30 striatum from Zeisel et. al[40] or **b)** versus the cell populations reported in Chen, et. al 2021[21], derived from adult mouse NAc. Correlation was performed on the combined top 100 markers/cell populations in which annotated homology exists. **c)** UMAP of cells colored by pseudotime values (from blue = early/immature to red = late/mature). The black trajectory line denotes the inferred differentiation path. **d)** Boxplot displaying the pseudotime distributions for each Interneuron (In) subtype starting from NPC. The early position of NB-1 supports its identification as an immature In subpopulation. **e)** Pseudotime-inferred expression dynamics of key neuronal markers for early (left panel) and late (right panel) pseudotime.

**Figure 5: Molecular mechanisms underpinning neuroblasts migration. a)** Representative 20x image of DAPI (blue) detection in a mouse brain section with annotated anatomical landmarks. LV, lateral ventricle; AC, anterior commissure, Dorsal Striatum. Scale bar, 500 um. **b)** Representative 20X images of DCX IHC in the NAc in adolescence (left panel) and adulthood (right panel). Scale bar, 100nm. **c)** Zoom-in image of DCX+ cells. Scale bar, 20um. **d)** Percentage of DAPI+ nuclei co-labeled with DCX within the NAc as quantified by CellProfiler. **e)** LIANA cell-to-cell interaction inference between NB-1(Target) and In-4 (source) neurons. Top highly-ranked interactions (adjusted rank <0.01) are shown. **f)** SCENIC *Nr2f1* regulon for In-4 interneurons showing *Nrf21* targets, some of which (yellow) are In-4 cell type marker genes.

**Supplementary Figure 1. Quality control metrics of snRNA-seq samples. a-c)** Violin plots displaying the number of detected genes (nFeature_RNA), total RNA counts (nCount_RNA), and percentage of mitochondrial gene counts (percent.mt) per individual sample. **d)** Number of high-quality nuclei captured per sample. **e-f)** Total nuclei counts separated by genotype and treatment condition.

**Supplementary Figure 2. Supplemental clustering and glutamatergic neuron identification. a)** UMAP visualization displaying additional clustering detail from snRNA-seq data. **b**) Expression of *Slc17a7*, a glutamatergic marker, highlighting potential glutamatergic populations within the NAc.

**Supplementary Figure 3. Feature plots of selected marker genes across NAc clusters.** UMAP visualizations show the expression patterns of key neuronal and glial marker genes used for cluster annotation: *Gad1, Foxp2, Drd1, Drd2, Drd3, Resp18, Top2a, Mbp, Pdgfra, Gja1, C1qa,* and *Vtn.*

**Supplementary Figure 4. UMAP visualizations demonstrating distribution by sample, genotype, and treatment.** Separate plots show nuclei distribution across clusters, colored by individual samples (top panel), genotype (middle panel), and treatment group (bottom panel), demonstrating even representation and integration across conditions.

**Figure Supplementary 5. Expression patterns of Reln across NAc cell types and genotype-dependent reduction of Reln. a)** Violin plots depicting expression levels of selected marker genes across medium spiny neurons (MSN) subtypes. **b)** Violin plots depicting reduced expression of Reln in specific neuronal populations (D1-MSN-1, D1-MSN-2, D2-MSN-2, GABA-1, GABA-4, and GABA-5) in heterozygous Reeler (Het, Reln+/−) mice compared to wild-type (WT) controls, confirming genotype-driven *Reln* haploinsufficiency. Values represent log-normalized gene expression. **c)** Violin plot depicting co-expression levels of *Gad1, Reln, Cnr1.*

**Supplementary Figure 6:** Heatmap of Pearson correlation values evaluating the relationship between our adolescent mouse-derived NAc cell classes (columns) versus the cell populations reported in Tran et al 2021, derived from human NAc. Correlation was performed on the combined top 100 markers/cell populations in which annotated homology exists.

**Supplementary Figure 7:** Volcano plots illustrating DEGs induced by THC exposure across Drd1-expressing medium spiny neurons (D1-MSNs), Drd2-expressing medium spiny neurons (D2-MSN), Interneurons (In) neurons, Neuroblasts (NB), and non-neuronal cell types in the adolescent NAc.

**Supplementary Figure 8:** Volcano plots illustrating DEGs induced by Reln haploinsufficiency Drd1-expressing medium spiny neurons (D1-MSNs), Drd2-expressing medium spiny neurons (D2-MSN), Interneurons (In) neurons, Neuroblasts (NB), and non-neuronal cell types.

**Supplementary Figure 9: Shared and distinct DEGs across cell types in the NAc following THC exposure and Reln haploinsufficiency. a)** Number of DEGs identified per cell type for THC treatment (top panel) and *Reln* genotype (bottom panel). **b)** Number of shared DEGs between THC treatment and Reln haploinsufficiency across cell types.

**Supplementary Figure 10: (a)** Number of modules with significant correlation with traits (genotype, treatment). **(b)** Heatmap of module-trait correlations between identified gene modules and experimental conditions (Treatment, Genotype). Color depicts the correlation coefficient *$p < 0.05$; **$p < 0.01$, ***$p < 0.001$. **(c)** Schematic of Mergeomics pipeline. MDF marker dependency filtering, MSEA marker set enrichment analysis, KDA key driver analysis.

**Supplementary Figure 11: a)** Diagram of the Custom CellProfiler Pipeline. **b)** Representative images of image set BBBC004v1 used to QC the customized CellProfiler pipeline. Each image contains exactly 300 computer-generated fluorescent objects but with an increasing probability of overlap (pOverlap; pOverlap of 0.3 indicates a 30% chance of overlapping). 20 images were generated for each pOverlap. **c)** Representative images showing segmentation of clustered fluorescent objects by the default pipeline and the customized pipeline. Outlines of identified objects from each pipeline were overlaid with the original image using the 'OverlayOutline' module of CellProfiler. The custom pipeline improved with over-segmentation and under-segmentation compared to the default pipeline. **d)** Disparity from ground truth (n=300 objects) was calculated for default and custom pipelines at different probabilities of overlap. Custom pipeline showed better performance as the number of objects identified was closer to ground truth.

## Acknowledgments

We thank Havilah Taylor and Patricia Montilla Perez for technical assistance with mouse colony management. This work was supported by NIH grants U01DA050239, DP1DA042232 to F.T. We acknowledge support by the UCSD Center for Epigenomics for generating snRNA-seq libraries and the UCSD IGM Genomics Center for sequencing the snRNA-seq libraries.

## Author contributions

F.T. conceived the project. A.I. performed drug treatments and generated tissue for snRNA-seq with assistance from A.T. Y.Z. performed bioinformatic analysis of snRNA-seq datasets with assistance from N.F. A.L. generated tissue for IHC experiments. D.S. performed IHC experiments with assistance from A.L. D.S developed the automated CellProfiler pipeline and performed statistical analysis of count data. All projects were supervised by F.T, and F.T. wrote the main text of the manuscript. Y.Z. and D.S. wrote the Materials and Methods section of the manuscript. All authors have approved the final version of the manuscript.

## Competing interests

The authors declare that they have no competing interests.
